# Patient Survey Exploring the Burden of Inflammatory Back Pain in Patients With Uveitis

**DOI:** 10.7759/cureus.37473

**Published:** 2023-04-12

**Authors:** Sian Bamford, Hasan Tahir, Zahra Ladan, Daren Hanumunthadu

**Affiliations:** 1 Physiotherapy, Royal Free London NHS Foundation Trust, London, GBR; 2 Medicine, University College London, London, GBR; 3 Rheumatology, Royal Free London NHS Foundation Trust, London, GBR; 4 General Medicine, Royal Free London NHS Foundation Trust, London, GBR; 5 Ophthalmology, Royal Free London NHS Foundation Trust, London, GBR

**Keywords:** quality improvement, delay to diagnosis, uveitis, inflammatory back pain, axial spondyloarthritis

## Abstract

Background

In the UK, diagnostic delays remain a challenge in axial spondyloarthritis (axSpA). Studies have shown that acute anterior uveitis is the most common extra-articular manifestation associated with axSpA. As part of a National Axial Spondyloarthritis Society (NASS) Aspiring to Excellence quality improvement project, this study aimed to ascertain the burden of inflammatory back pain (IBP) in patients attending a uveitis clinic and to establish the number of these patients who had not been referred to a rheumatologist, thereby contributing to the diagnostic delay. The secondary aims were to explore the factors contributing to the diagnostic delay.

Methods

A 22-question patient survey was created to identify the burden of back pain in patients attending a specialist uveitis clinic at a London NHS Trust. Participants were recruited when attending their clinic appointments. Survey content included patient demographics and whether they had experienced back pain for longer than three months. The Berlin Criteria was used to identify the presence of inflammatory back pain, and it was also ascertained whether participants had a previous diagnosis of axSpA. Participants were asked if they had seen any healthcare professionals regarding their back pain and the total number of consultations they had had with each profession.

Results

A cohort of 50 patients who attended the uveitis clinic at the Royal Free London NHS Trust completed the survey between February and July 2022.

The mean age of the respondents was 52 years with a mean length of time with uveitis of 6.57 years. Of them, 64% were female and 36% were male. Forty per cent (40%) of participants (20 respondents) reported experiencing back pain for more than three months and 12% (six respondents) had a diagnosis of axSpA.

Of those who reported back pain for more than three months, the mean age of onset of back pain was 28.6 years. Of the 14 participants (28%) who had back pain and were not diagnosed with axSpA, nine (18%) fulfilled the Berlin criteria for IBP.

All participants had seen a GP or allied health professional specifically for their back pain. On average, respondents had seen two allied healthcare professionals, but only 40% (eight) of respondents with back pain had been seen by a rheumatologist.

Conclusions

In this study, the data highlights that inflammatory back pain is common in patients with uveitis and the majority of patients with inflammatory back pain had not been referred to a rheumatology service and potentially have undiagnosed axSpA. Contributing factors to this potential delay in diagnosis include a lack of awareness of axSpA and its presenting features and associated conditions and a lack of onward referral for a specialist rheumatology opinion. This highlights the need for public, patient and healthcare professional education and the development of timely referral pathways to reduce delays in diagnosis.

## Introduction

Introduction 

In the United Kingdom, there is an 8.65-year delay in the diagnosis of axial spondyloarthritis (axSpA) [1]. This delay can result in higher disease activity, worse physical function and increased structural damage [2,3]. Diagnostic delay is also associated with an increased likelihood of depression and reduced quality of life at a time in life when individuals are trying to establish careers, relationships and families [3,4]. Patient factors, such as female sex, negative HLA-B27 and previous mechanical back pain, have been shown to be associated with a prolonged time to diagnosis [3,5].

Acute anterior uveitis is the most common extra-articular manifestation associated with axSpA, occurring in 25-35% of patients [6]. The aim of this study was to explore the prevalence of inflammatory back pain (IBP) in a uveitis clinic and ascertain the number of patients who had been diagnosed with axSpA and were under specialist care, and how many remained potentially undiagnosed. Wider aims included evaluating whether any improvement in referral pathways for patients with IBP to specialist rheumatology services was required and if there was a need to increase awareness of axSpA among uveitis patients and healthcare professionals to improve identification of IBP and reduce the time to diagnosis. This study was part of a National Axial Spondyloarthritis Society (NASS) Aspiring to Excellence quality improvement project [7].

## Materials and methods

A prospective analysis was undertaken of patients in a London NHS trust from February to July 2022. Participants were recruited following attendance at the uveitis clinic. After an explanation of the aim of the survey, verbal consent was taken, and a 22-question survey was completed. 

The survey included patient demographics, the length of time they had had uveitis and whether participants had previously been diagnosed with axSpA. The survey established whether participants were currently taking or had previously taken a biologic medication. Participants were asked if they had experienced back pain lasting over three months, and if so, their age at symptom onset, the location of symptoms and whether their symptoms improved with non-steroidal anti-inflammatory medication. The presence of IBP was identified using the Berlin Criteria. The Berlin Criteria confirms IBP in individuals that have experienced back pain for more than three months, are under the age of 50 years and fulfil at least two of the following criteria: morning stiffness >30 minutes, improvement of back pain with exercise but not rest, nocturnal awakening during the second half of the night and alternating buttock pain [8]. 

The full questionnaire is detailed in the Appendices.

## Results

A total of 121 patients were approached, of which 50 consented to complete the survey. Forty per cent (40%) of participants (20) reported that they had experienced back pain for more than three months. Twelve per cent (12%) of participants (6) had an established diagnosis of axSpA. Participant demographics and key results are presented in Table 1.

**Table 1:** Summary of participant demographics and key results of the survey

Measure	Result
Age on completing the survey, years (mean)	52
Sex, female (%, participants)	64%, 32
Length of time with uveitis, years (mean)	6.57
Back pain for > 3 months (%, participants)	40%, 20
Diagnosis of axSpA (%, participants)	12%, 6

Of the remaining 14 participants (28%) who had back pain and were not diagnosed with axSpA, six females (12%) and three males (6%) fulfilled the Berlin Criteria for IBP (Table 2). Two individuals, one male and one female, within this subgroup had a previous diagnosis of psoriasis and two participants, both female, had a previous diagnosis of inflammatory bowel disease (Table 2).

**Table 2:** Presence of the features of the Berlin Criteria and the extra-articular manifestations of axSpA in the 14 participants with back pain without a previous diagnosis of axSpA axSpA: axial spondyloarthritis

Measure	Results (%, participants)
Improvement with activity	43%, 6
Morning stiffness > 30 mins	71%, 10
Waking in the second half of the night with pain	79%, 11
Alternating buttock pain	43%, 6
Psoriasis	14%, 2
Inflammatory bowel disease	14%, 2

**Figure 1:**
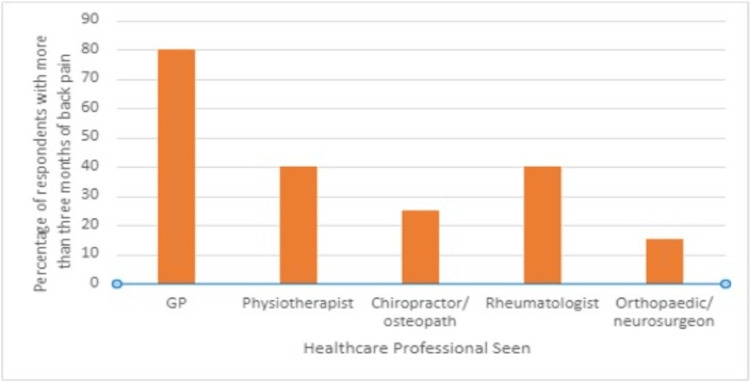
Graph illustrating the healthcare professionals seen by patients reporting more than three months of back pain

Only eight of the 20 respondents that had experienced back pain for longer than three months had seen a rheumatologist. Figure 1 illustrates which healthcare practitioners' participants had seen specifically for their back pain symptoms. 

## Discussion

Acute anterior uveitis is the most common extra-articular manifestation associated with axSpA, occurring in 25-35% of patients [6]. Studies have shown that the presence of uveitis has a mixed impact on the time to diagnosis of axSpA. In 2022, Hay et al. conducted a systemic review exploring the factors associated with a delay in diagnosis, and they identified five studies that assessed the impact of the presence of uveitis - three studies showed no association, one showed an increase in time to diagnosis and one showed a decrease in time to diagnosis [9]. Despite this, there is evidence that there is a significant burden of IBP and potentially undiagnosed axSpA in patients presenting with uveitis. We found that 40% of uveitis patients attending a clinic experienced back pain for longer than three months and 12% had an established diagnosis of axSpA. Twenty-eight per cent (28%) of participants had back pain without any formal diagnosis and of these, 64% fulfilled the Berlin Criteria and therefore may have been undiagnosed as axSpA. This is in keeping with the results of the multicentre study conducted by Weber et al. They used clinical features, biochemical markers and imaging to diagnose axSpA in 61.6% of acute anterior uveitis patients [10].

Barnett et al. explored the reasons for the delay in diagnosis of axSpA and highlighted that a lack of referrals to rheumatologists, from both primary and secondary care services, played a significant role [11]. Our results echo their findings; all participants experiencing IBP had seen their GP or another healthcare professional specifically for their back pain, however, only 40% had seen a rheumatologist. This highlights a lack of onward referral for a specialist opinion and a potential delay in diagnosis for some individuals. Several studies have been conducted to explore the reason for the lack of onward referral. Jois et al. conducted a survey and found that only 5% of GPs were able to recognise the eight key features of IBP [12]. This suggests that the lack of referrals is in part due to a lack of awareness of IBP and improving education in primary care may improve IBP recognition and increase the number of referrals for a specialist opinion [12]. In this study, of the nine patients with back pain that fulfilled the Berlin Criteria, four individuals had a previous diagnosis of either psoriasis or inflammatory bowel disease alongside their diagnosis of uveitis, but only one of these participants had seen a rheumatologist regarding their persistent back pain. These findings highlight the need for increased awareness of the conditions linked to axSpA among patients and clinicians. Improving public education and awareness has been shown to have a positive impact on referral rates. In New Zealand, a public awareness campaign focusing on chronic back pain and the features of IBP resulted in an increase in referrals and an increase in the number of patients diagnosed with axSpA [13].

Further work must also be carried out to improve awareness of axSpA in secondary care and to simplify internal referral pathways. The DUET study carried out by Haroon et al. concluded that approximately 40% of patients presenting to an ophthalmology hospital emergency department with anterior uveitis had undiagnosed SpA, which supports the results of our study [14]. Rademacher et al. proposed that all anterior uveitis patients with musculoskeletal symptoms should have a rheumatological evaluation due to the high prevalence of SpA among this cohort and the high number of cases that are undiagnosed [15]. Gaffney et al. suggested that educational campaigns for clinicians that start during their undergraduate training may be of benefit. They also proposed utilising electronic hospital records to flag onward referral for a specialist rheumatological opinion when patients with acute anterior uveitis, inflammatory bowel disease or psoriasis experience symptoms of IBP [16].

Limitations 

The major limitation of this study was the small sample due to difficulties in recruiting participants who had attended the uveitis clinic. Additionally, the study included participants with all types of uveitis. We acknowledge that, as with all survey-based studies, there is an element of recall bias.

## Conclusions

The work carried out in this study and by NASS recognises the need to reduce the time to diagnosis of axSpA and seeks to improve the quality of life of patients with axSpA. IBP is not identified and is therefore underdiagnosed in patients with uveitis despite persistent back pain being prevalent in these patients. The lack of awareness of IBP leads to a delay in referrals to secondary care rheumatology services and increases the time to diagnosis and treatment. Improving education amongst primary care clinicians and ophthalmologists will increase the awareness of axSpA and aid identification and onward referral of patients with potential axSpA and ultimately reduce the delay in diagnosis. Moreover, a collaboration between ophthalmologists and rheumatologists will allow for the development of better internal referral pathways, increasing the number of referrals for a specialist rheumatological opinion and the potential diagnoses of axSpA. 

## Appendices

**Table 3:** The 22-question survey used in this study

1	Have you been diagnosed with inflammatory back pain/ankylosing spondylitis/axial spondyloarthritis?*							
	Yes			No				
2	What is your date of birth?*							
3	What is your gender?*							
	Male	Female	Other		Prefer not to say			
4	How long (in years) have you had uveitis for?*							
5	Are you currently taking biologic medication?*							
	Yes		No					
6	If yes – Which biologic are you currently taking? Free text							
7	If no – Have you ever taken biologic medication?							
	Yes		No					
8	Do you experience back pain that has lasted for longer than 3 months?*							
	Yes		No					
9	If yes - Does it improve with NSAIDs i.e. ibuprofen, Naproxen?							
	Yes		No					
10	How old were you (in years) when your back pain started?*							
11	Where is your pain located? (Select all relevant)*							
	Neck	Mid back	Lower back				N/A	
12	Does your back pain improve with activity or rest?*							
	Activity	Rest	Neither				N/A	
13	Have you ever seen a health care professional regarding your lower back pain?*							
	Yes		No					
14	If yes – Which healthcare professionals have you seen? (Select all relevant)							
	GP	Physiotherapist	Chiropractor/ osteopath			Orthopaedic/ neurosurgeon		Rheumatologist
15	Do you experience morning stiffness lasting longer than 30 minutes?*							
	Yes		No					
16	Do you wake during the second half of the night due to back pain?*							
	Yes		No					
17	Do you have alternating buttock pain?*							
	Yes		No					
18	Do you experience pain and swelling in other joints?*							
	Yes		No					
19	Have you ever had plantar fasciitis (pain in the heel/ arch of your foot) or Achilles tendinitis (pain in the tendon at the back of your ankle)?*							
	Yes		No					
20	Have you ever had psoriasis?*							
	Yes		No					
21	Have you ever had inflammatory bowel disease?*							
	Yes		No					
22	Does anyone in your family have Ankylosing Spondylitis?*							
	Yes		No					
